# Weak coupling of neurons enables very high-frequency and ultra-fast oscillations through the interplay of synchronized phase shifts

**DOI:** 10.1162/netn_a_00351

**Published:** 2024-04-01

**Authors:** Lenka Přibylová, Jan Ševčík, Veronika Eclerová, Petr Klimeš, Milan Brázdil, Hil G. E. Meijer

**Affiliations:** Department of Mathematics and Statistics, Faculty of Science, Masaryk University, Brno, Czech Republic; Institute of Scientific Instruments, The Czech Academy of Sciences, Brno, Czech Republic; Brno Epilepsy Center, Dept. of Neurology, St. Anne’s Univ. Hospital and Faculty of Medicine, Masaryk University, Brno, Czech Republic, member of the ERN EpiCARE; Behavioral and Social Neuroscience Research Group, Central European Institute of Technology, Masaryk University, Brno, Czech Republic; Department of Applied Mathematics, Techmed Centre, University of Twente, Enschede, The Netherlands

**Keywords:** Very high-frequency oscillations, Ultra-fast oscillations, Neuronal network model, Phase-shift synchrony, Bifurcations, Epilepsy

## Abstract

Recently, in the past decade, high-frequency oscillations (HFOs), very high-frequency oscillations (VHFOs), and ultra-fast oscillations (UFOs) were reported in epileptic patients with drug-resistant epilepsy. However, to this day, the physiological origin of these events has yet to be understood. Our study establishes a mathematical framework based on bifurcation theory for investigating the occurrence of VHFOs and UFOs in depth EEG signals of patients with focal epilepsy, focusing on the potential role of reduced connection strength between neurons in an epileptic focus. We demonstrate that synchronization of a weakly coupled network can generate very and ultra high-frequency signals detectable by nearby microelectrodes. In particular, we show that a bistability region enables the persistence of phase-shift synchronized clusters of neurons. This phenomenon is observed for different hippocampal neuron models, including Morris–Lecar, Destexhe–Paré, and an interneuron model. The mechanism seems to be robust for small coupling, and it also persists with random noise affecting the external current. Our findings suggest that weakened neuronal connections could contribute to the production of oscillations with frequencies above 1000 Hz, which could advance our understanding of epilepsy pathology and potentially improve treatment strategies. However, further exploration of various coupling types and complex network models is needed.

## Author Summary

We have built a mathematical framework to examine how a reduced neuronal coupling within an epileptic focus could lead to very high-frequency (VHFOs) and ultra-fast oscillations (UFOs) in depth EEG signals. By analyzing weakly coupled neurons, we found a bistability synchronization region where in-phase and anti-phase synchrony persist. These dynamics can be detected as very high-frequency EEG signals. The principle of weak coupling aligns with the disturbances in neuronal connections often observed in epilepsy; moreover, VHFOs are important markers of epileptogenicity. Our findings point to the potential significance of weakened neuronal connections in producing VHFOs and UFOs related to focal epilepsy. This could enhance our understanding of brain disorders. We emphasize the need for further investigations of weakly coupled neurons.

## INTRODUCTION

It is known that hippocampal pyramidal neurons can switch from integrators to resonators, that is, from class I excitability to class II (Prescott, Ratté, De Koninck, & Sejnowski, 2008). This dual operating mode allows for individual spikes, tonic spiking, and bursting, as well as different firing rates (Ermentrout & Terman, 2010; Izhikevich, 2006). Specifically, there is a maximum firing frequency above which the potential on the neuron membrane cannot oscillate faster; oscillations occur at a certain natural frequency for a given external stimulus. Although different types of neurons have different rates of action potential firing, these are physiologically limited (Gabbiani & Cox, 2017; Purves et al., 2019). The *f*–*I* curve (frequency given a fixed input *I*) typically saturates to a finite level not much above 350 Hz, even for the fastest spiking neurons; see Wang et al. (2016). Using mathematical conductance-based models, we can explain the onset and offset of the frequency–input curve. For the offset, one typically encounters a Hopf bifurcation or a limit point of cycles (Izhikevich, 2006; Kuznetsov, 2023). Biophysically plausible parameter settings then show we cannot expect higher frequencies. The dependence of the individual neuron model dynamics on the external current and other parameters has been studied extensively (Cessac & Samuelides, 2007; Duan & Lu, 2006; Shilnikov, 2012; Tsumoto, Kitajima, Yoshinaga, Aihara, & Kawakami, 2006; Xing, Song, Wang, Yang, & Chen, 2022). This biophysical basis of the mechanism is also confirmed by comparison with a real neuronal signal (Prescott, De Koninck, & Sejnowski, 2008).

Nevertheless, hippocampal electroencephalographic signals (EEG) show that high-frequency oscillations (HFOs) are reported in the seizure onset zone and are connected with epileptic seizure generation in humans with focal epilepsy (Cimbalnik et al., 2018, 2020; Jacobs et al., 2008, 2012; Jiruska et al., 2017; Pail et al., 2020; Řehulka et al., 2019; Staba & Bragin, 2011; Worrell & Gotman, 2011). Moreover, the extent of HFOs correlates with seizure frequency and disease severity (Zijlmans, Jacobs, Zelmann, Dubeau, & Gotman, 2009). HFOs are most commonly distinguished by frequency bands as interictal high gamma (65–100 Hz), ripples (100–250 Hz), and fast ripples (250–600 Hz). Recently, very high-frequency oscillations (VHFOs, 600–2000 Hz; Brázdil et al., 2017; Travnicek et al., 2021; Vasickova et al., 2023), very fast ripples (VFRs, 600–1000 Hz), and ultra-fast ripples (UFRs, 1000–2000 Hz), and most recently ultra-fast oscillations (UFOs, over 2000 Hz) were reported in depth EEG recordings of patients with epilepsy (Brazdil et al., 2023). Since VHFOs were more spatially restricted in the brain than HFOs with lower frequencies, they have been suggested as novel biomarkers of the epileptogenic zone. Moreover, VFRs present intriguing synchronization phenomena, demonstrating strong phase-locking with slow oscillations (Hao et al., 2021). This highlights the crucial role synchronization mechanisms may play in the pathological progression and underlying propagation of epileptiform discharges within the hippocampal network during status epilepticus in temporal lobe epilepsy. However, the neurons themselves are unable to produce such high oscillation frequencies. This brings us to the question of whether multiple coupled neurons are capable of producing a synchronized signal of such high frequency.

It is known that synchronization occurs in oscillators for sufficiently strong coupling (Boccaletti, Pisarchik, Del Genio, & Amann, 2018; Pikovsky, Rosenblum, & Kurths, 2001; Strogatz, 2018; Wiggins, 2003) and even that it can occur through external noise (Lang, Lu, & Kurths, 2010; Nagai & Kori, 2010; Zhou & Kurths, 2002). At the same time, the dynamics of coupled oscillators may be complex, chaotic, or consist of chimera states (Abrams & Strogatz, 2004; Aihara, Takabe, & Toyoda, 1990; Calim, Hövel, Ozer, & Uzuntarla, 2018; Hoppensteadt & Izhikevich, 1997; Majhi, Bera, Ghosh, & Perc, 2019). Therefore, it is important to determine how coupled neuron models behave dynamically for physiological parameters and whether and, if so, how they can be synchronized with higher than natural frequencies, especially within VHFO and UFO frequency bands.

Within this paper, we consider near-identical neurons weakly connected through gap junctions. In this setup, we show that local field potentials can exhibit VHFOs and UFOs through phase synchronization. Moreover, we claim that it is not an exceptional phenomenon but quite the opposite in the case of very weak neuronal couplings. It arises for various connectivity types and neuron models. The mechanism is based on theoretical works on the symmetry of dynamical networks that can also be spatiotemporal and can lead to a rigid phase-shift synchrony (Golubitsky, Messi, & Spardy, 2016; Golubitsky, Romano, & Wang, 2012; Golubitsky & Stewart, 2006, 2016; Golubitsky, Stewart, & Török, 2005). We show that the occurrence of these higher frequencies is possible both in the case of different values of model parameters and in the case of random noise affecting the magnitude of the external current input.

In the Results section, we first discuss the Morris–Lecar neuron model, which we examine thoroughly. This includes a discussion of its fundamental dynamics, followed by an exploration of Morris–Lecar neuronal networks. We then analyze the behavior of two coupled Morris–Lecar neuron models, explain the phase shift, and reveal the concept of anti-phase collective synchrony in a model of a neuronal network. We show that we can achieve transient anti-phase synchrony by stimulating a subnetwork of neurons due to the existence of the bistability region. This concept can lead to an apparently higher frequency in the EEG signal. We want to demonstrate that the observed phenomena of phase-shift synchrony and bistability are generic for weak coupling and still hold for more physiological models of neurons. The analysis of the Morris–Lecar model is straightforward as the single-neuron model is two-dimensional. The hippocampal neuronal network, however, is considerably more complex than the simple networks composed of Morris–Lecar neuron models, which exhibit a specific course of spike dynamics. We show that the bistability and anti-phase synchronization appear robustly in network models with more complex neuronal dynamics. One model is an interneuron network that exhibits VHFOs and UFOs with anti-phase locking, and the other is a Destexhe–Paré neuron network that shows VHFOs transiently. Similarly to the Morris–Lecar model, investigating the dynamics of two coupled interneurons, we find regions with synchronization and bistability and present simulations of the collective anti-phase behavior. We discuss transient ultra-fast ripples and ultra-fast oscillations in a two-level neuronal network. Lastly, simulations of the collective anti-phase behavior are presented for the Destexhe–Paré neuron model. In the Discussion and Conclusions section, we detail our primary objective to investigate whether weakened connections between neurons in an epileptic focus could result in high, very high-frequency, and ultra-fast oscillations; we examine how our findings support this theory and how they provide a mathematical framework for interpreting EEG signals from patients with focal epilepsy, while also highlighting the need for future research on the effects of different coupling types, strengths, and parameters on the dynamics of coupled neurons.

## RESULTS

### Model Dynamics Explaining the Tonic Spiking

**Figure 1.**
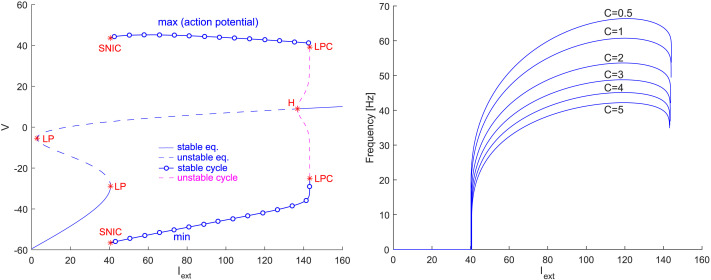
Morris–Lecar model dynamics with respect to the applied external current *I*_ext_. *Left*: The bifurcation diagram shows a limit point (LP) for *I*_ext_ ≈ 40 where a saddle-node on an invariant curve (SNIC) leads to a stable cycle, which exists for a wide range of external current values (tonic spiking region) until the limit point of cycles near *I*_ext_ = 140. The branch of cycles turns and becomes unstable, and ends at a subcritical Hopf bifurcation (H). Beyond the LPC, neuronal excitation is impossible. The other parameter values are listed in Table 1, and the membrane capacitance is *C* = 5 for simplicity. *Right*: The natural firing frequency as a function of external current *I*_ext_ for various membrane capacitances *C*. As the capacitance increases, only the frequency of the oscillation decreases but does not affect the existence of the limit cycle. Other parameter values are listed in Table 1.

Let us recall basic neuron spiking mechanisms that lead to tonic spiking clearly described in Prescott, De Koninck, and Sejnowski (2008). Class I neurons continuously increase their firing rate in response to a steady increase in input current without any abrupt change in their behavior. This type of behavior arises from a saddle-node on an invariant curve (SNIC) bifurcation, characterized by a break of a limit cycle on a saddle-node equilibrium as the input current is varied. On the contrary, class II neurons exhibit a discontinuous increase in their firing rate in response to a change in the input current, with a threshold for tonic spiking emergence. This behavior occurs near a subcritical Hopf bifurcation when a stable spiking orbit already exists due to a saddle-node of limit cycles with respect to the input current. The bifurcation diagram presented in Figure 1 demonstrates that the Morris–Lecar system Equation 11 shows both bifurcations at onset and offset as we vary the external input external current *I*_ext_, as mentioned. Note that class III neurons do not possess tonic spiking dynamics with a given natural frequency; hence, we will not focus on their type of mechanism. From now on, we will assume the neuron exhibits tonic spiking dynamics with a certain given natural frequency under a suitable external stimulus current.

External current and parameter changes influence the neuron natural frequency; see Figure 1. Membrane capacitance depends on the cell’s and membrane’s biophysical properties, including fluidity, thickness, permeability, and so forth. (Bakhtiari, Manshadi, Candas, & Beskok, 2023). Moreover, recently published studies (Patel, Tewari, Chaunsali, & Sontheimer, 2019; Tewari et al., 2018) propose pathological changes in the effective capacitance of neurons that may contribute to epilepsy. Therefore, the capacitance appears to be an appropriate parameter for the bifurcation analysis in relation to the emergence of HFOs, VHFOs, and UFOs in epilepsy patients. Similarly, one can investigate the dependence on other model parameters influencing the neuron frequency, for example, *φ* and *I*_ext_ (e.g., see Liu, Liu, & Liu, 2014; Tsumoto et al., 2006; Xing et al., 2022) or *g*_*Ca*_, *g*_*K*_, and *g*_*L*_ that can also be influenced by drugs (see Macdonald, 1989; Nicholson, Blanche, Mansfield, & Tran, 2002).

### Two Coupled Morris–Lecar Neuron Models

**Figure 2.**
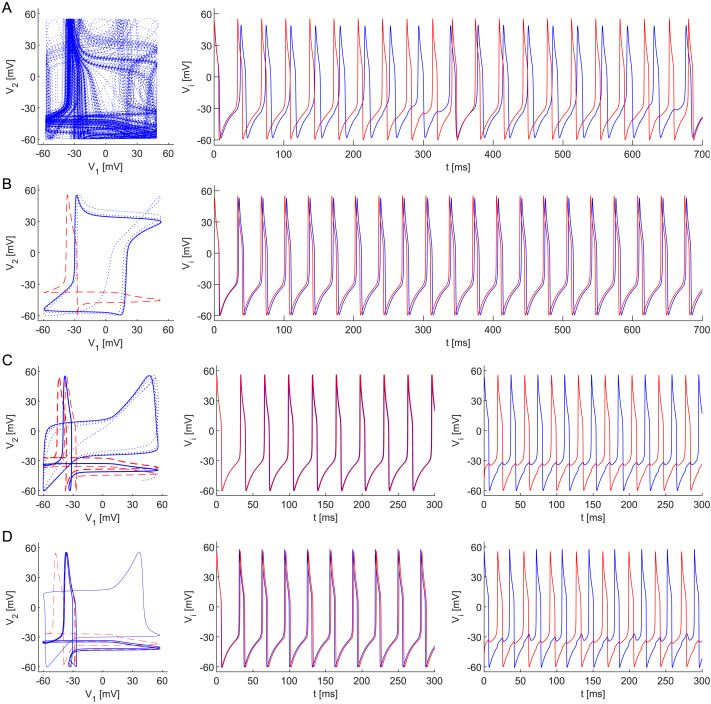
Dynamics in the system (Equation 15) for *ε* = 0.05, various values of *C*_1_, and fixed *C*_2_ = 1 (see Figure 4). *Left*: projections of orbits (blue dotted line), and stable (blue solid line) and unstable (red dashed line) cycles onto the state space *V*_1_ × *V*_2_ found by continuation. *Right*: time course of *V*_1_ (blue) and *V*_2_ (red) of the blue orbits in the left panel. Other parameter values are listed in Table 1. Choosing *C*_1_ affects the dynamics: (A) *C*_1_ = 2.75, quasi-periodicity. (B) *C*_1_ = 1.60, a stable in-phase periodic solution (IPS; the bold blue cycle on the left state space *V*_1_ × *V*_2_). (C) *C*_1_ = 0.90, bistability, the system possesses stable in-phase and anti-phase periodic solutions (IPS and APS; the bold blue cycles on the left state space *V*_1_ × *V*_2_). (D) *C*_1_ = 0.581, bistability, anti-phase cycle lost stability through a period-doubling bifurcation (PD).

To get insight into the behavior of a large Morris–Lecar neuronal network, let us focus on the smallest possible network first. Consider a pair of bidirectionally coupled Morris–Lecar neurons (Equation 15) for *N* = 2. It is well known that if the ratio of the frequencies of two oscillators is irrational, the orbit lives on a two-dimensional invariant torus and is called asynchronous or quasi-periodic (Wiggins, 2003). For a strong enough coupling *ε* between them, the dynamics of both oscillators can become mutually synchronized. If the phases of two synchronized oscillators are similar or opposite, we refer to this as in-phase (IPS, Figure 2B) and anti-phase synchrony (APS, Figure 2C), respectively; see Pikovsky et al. (2001). For very different frequencies, even strong coupling might not be capable of synchronizing the dynamics of the coupled systems, see Figure 2A, while for close frequencies, the dynamics are quasi-periodic only for very weak coupling.

### Phase Shift for Two Weakly Coupled Morris–Lecar Neuron Models

Two identical oscillators form a symmetrical dynamical system with a symmetrical periodic solution. The symmetry of the system is roughly preserved even for non-identical oscillators due to the continuous dependence of the solutions on parameters (see Perko, 2001), that is, coupled neurons with similar phase dynamics form a nearly symmetrical system with a stable limit cycle such that the phases of both tonically spiking neurons are mutually shifted, although having a common frequency roughly the magnitude of the natural frequencies of the individual neurons. This phenomenon, so-called phase-locking, and the theoretical background for this mechanism is based on Golubitsky and Stewart’s results of equivariant bifurcation theory allowing groupoid formalism for networks of systems of differential equations (Golubitsky et al., 2012, 2016; Golubitsky & Stewart, 2006, 2016; Golubitsky et al., 2005; Nijholt, Rink, & Sanders, 2019). The symmetry of the network with a *T*-periodic solution implies the existence and persistence of a rigid phase-shift synchrony. In our case of two nearly identical weakly coupled oscillators (Equation 15), there may also exist solutions synchronized with time shift *T*/2 for appropriate system parameters: the attracting stable cycle, corresponding to the presence of an anti-phase synchronous state (see Figure 2C). Simultaneously, the system possesses a solution reflecting in-phase synchrony where the neurons oscillate almost identically; see Figures 2B, 2C (middle), and 2D (middle).

**Figure 3.**
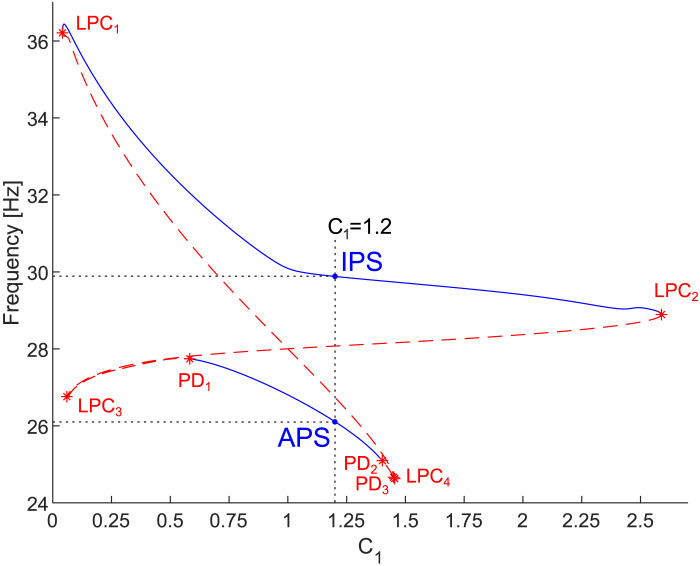
Frequency of the limit cycles in the system (Equation 15) of two coupled Morris–Lecar neuron models with respect to *C*_1_. The remaining parameter values are identical (see Table 1), *C*_2_ = 1, and *ε* = 0.05. The upper and bottom blue branches correspond to the stable in-phase and anti-phase synchrony, respectively. The dashed sections at the given parameter values in Figure 4 correspond to these depicted branches. Notice the bistability, present for a relatively wide range of *C*_1_. For completeness, we remark that there exist more and more complex periodic solutions of the system (Equation 15) for *C*_1_ between the LPC_3_ and PD_1_ points, and between PD_2_ and PD_3_ points that are not depicted in this figure. The dashed line at *C*_1_ = 1.2 denotes the frequencies at IPS near 30 Hz and APS near 26 Hz that reappear in the simulations depicted in Figures S1 and S2 in the Supporting Information.

**Figure 4.**
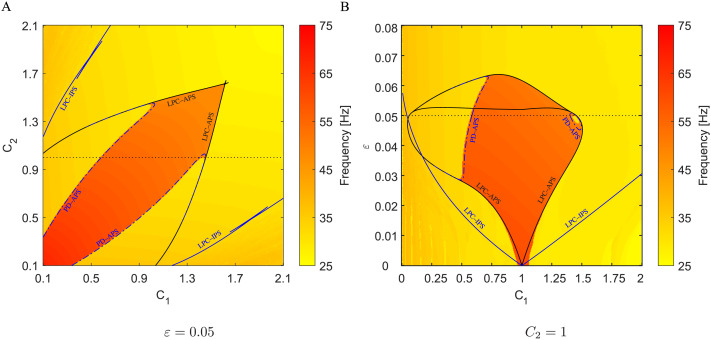
Arnold tongues in parameter spaces (A) *C*_1_ × *C*_2_ and (B) *C*_1_ × *ε* for two coupled Morris–Lecar neuron models (Equation 15). The regions are delineated by blue and black solid LPC curves corresponding to the IPS and APS, respectively. The blue dash-dotted curves refer to PD of the anti-phase cycle. The background color indicates the dominant frequency in a simulated composed signal *V* = *V*_1_ + *V*_2_. In the region bounded by the PD curves, the system may exhibit more complex, even chaotic dynamics. The red region corresponds to the stable anti-phase cycle, while the other solutions (yellow) have a frequency close to the natural frequency. Compare with Figure 2: (A) quasi-periodicity outside Arnold tongues; (B) IPS inside the outer Arnold tongue; (C) bistability in the red region delineated by LPC curves and PD curves; (D) APS lost stability due to PD, frequency halves.

Both branches of stable periodic solutions, that is, in-phase and anti-phase synchrony, persist inside relatively wide regions of the parameter space; see Figure 3. This fact is also depicted in Figure 4 where the bifurcation diagrams with respect to *C*_1_ and *C*_2_, and *ε* and *C*_1_, respectively, are shown. More accurately, Figure 3 precisely corresponds to the dashed sections at *C*_2_ = 1 and *ε* = 0.05 in Figure 4. The regions, so-called Arnold tongues or resonance tongues (Kuznetsov, 2023; Wiggins, 2003), are delineated by borders belonging to the limit point of cycle (LPC) manifolds. Outside the Arnold tongues, the difference in capacitances *C*_1_ and *C*_2_ implies a synchronous state with heterogeneous frequencies (see Figure 1), quasi-periodic oscillations, or more complex dynamics. The LPC manifolds were computed for parameters listed in Table 1. Since interchanging *C*_1_ and *C*_2_ mirrors the synchronization regions, the black and blue solid curves in Figure 4A are symmetrical. Cusps in the intersections of two branches of LPC manifolds are co-dimension two bifurcation points (Kuznetsov, 2023). Although the LPC curves in Figure 4 were computed for given parameters from Table 1, the generality of bifurcation theory guarantees that these LPC manifolds persist for nearby parameter values.

**Table 1.** Typical physiological parameter values for the Morris–Lecar neuron model of a hippocampal pyramidal cell (Gutkin & Ermentrout, 1998)

	Setting	Unit	Meaning
*C*	0.1–2.6	*μ*F/cm^2^	membrane capacitance
*g*_L_	2	mS/cm^2^	maximum leak conductance
*g*_Ca_	4	mS/cm^2^	maximum Ca^2+^ conductance
*g*_K_	8	mS/cm^2^	maximum K^+^ conductance
*V*_L_	−60	mV	equilibrium potential of leak channel
*V*_Ca_	120	mV	equilibrium potential of Ca^2+^ channel
*V*_K_	−80	mV	equilibrium potential of K^+^ channel
*β*_1_	−1.2	mV	tuning parameters for Ca^2+^ activation function
*β*_2_	18	mV	
*β*_3_	10	mV	tuning parameters for K^+^ activation function and time function *τ*_*w*_
*β*_4_	17.4	mV	
*φ*	1/15	s^−1^	reference frequency
*I*_ext_	43	*μ*A/cm^2^	externally applied direct current

Similarly, the bifurcation diagram in Figure 4B shows the Arnold tongues with respect to the coupling strength *ε* and membrane capacitance *C*_1_. In this case, they emanate from the point *C*_1_ = *C*_2_ (other parameters are identical) since the limit case *ε* = 0 belongs to the uncoupled pair of neurons with identical frequencies. Generally, nonidentical similar neurons will have similar intrinsic frequencies, and the Arnold tongue would emanate from a cusp point with the same frequency, but the membrane capacitances *C*_1_, *C*_2_, as well as other parameters, would differ slightly. Moreover, results presented in Kobelevskiy (2008), which deals with Morris–Lecar models that incorporate time-delayed gap-junctional coupling, suggest that similar dynamics will emerge in such models. Furthermore, an increase in delay may even play a stabilizing role.

The background color in the bifurcation diagrams in Figure 4A and 4B indicates the dominant frequency detected in a composed signal *V* = *V*_1_ + *V*_2_ simulated on a time interval [200, 1,200], discarding 200 ms transients, for parameter values from a 401 × 401 grid and appropriate initial conditions. These regions may not match precisely since the two-cycle created by crossing the PD curves has a similar spike shape to the cycle corresponding to the APS (see Figure 2D). Furthermore, around the point [1, 0] in Figure 4B, that is, for very weak coupling and similar membrane capacitances, the observed dominant frequency of ∼60 Hz in the composition is caused by long-lasting transient dynamics.

### Anti-Phase Synchrony in a Model of Morris–Lecar Neuronal Network Leading to Higher Collective Frequency

A similar approach can be used for more complex networks of (nearly) identical neurons with symmetry. The phenomenon leads to complete collective synchrony or phase-shift synchrony in the network of neurons.

Experiments, simulations, and theory show that anti-phase collective synchronization is possible (Chowdhury, Rakshit, Buldu, Ghosh, & Hens, 2021; Kawamura, Nakao, Arai, Kori, & Kuramoto, 2010; Sebek, Kawamura, Nott, & Kiss, 2019). Ermentrout and Terman (2010) previously reported on the anti-phase dynamics of two Morris–Lecar neurons with synaptic coupling; however, our findings demonstrate that this phenomenon is not exceptional and has the potential to lead to a rise of higher frequency in the summed signal. Furthermore, we have observed similar collective dynamics in a network, highlighting the robustness of this phenomenon across different scales of neuronal systems.

Consider a neuronal network model (Equation 15) of *N* = 50 weakly coupled Morris–Lecar neurons. Based on the results presented in the previous subsection, one can expect the presence of multistability in this system, that is, the coexistence of multiple stable solutions corresponding to various synchronous states depending on the initial conditions and varied model parameters *ε* and *C*_*i*_, as is depicted in Figures S1 and S2 in the Supporting Information.

Specifically, for the demonstration of the collective anti-phase behavior, let us suppose identical coupling strength *ε* = 0.1/50 = 0.002. Further, we will consider that the heterogeneity of neurons results from differences in their membrane capacitances (one may assume similar heterogeneity in other parameters, as mentioned before). Let *C*_*i*_ be independent and follow a truncated normal (TN) distribution defined on the interval [0.75, 1.05] to avoid unrealistic or extreme values, that is, *C*_*i*_ ∼ TN(0.90, 0.05^2^, 0.75, 1.05), while the remaining parameters stay identical (see Table 1). We base our choice for the mean and standard deviation of neuron membrane capacitances *C*_*i*_ on direct measurements from Gentet, Stuart, and Clements (2000). To account for possible pathological changes near the epileptic focus, we also explored high values of the standard deviation. Finally, let the population consist of two subpopulations of roughly the same size, 𝒫_1_ = {1, …, 25}, 𝒫_2_ = {26, …, 50}, that have random initial conditions that partially overlap

$$V_{i}\left(0\right)∼\text{TN}\left(-35,5^{2},-50,-20\right),i\in {𝒫}_{1}\cup {𝒫}_{2},w_{i}\left(0\right)∼\left\{\begin{array}{ll} \text{TN}\left(0.01,0.025^{2},0,0.085\right), & i\in {𝒫}_{1},\\ \text{TN}\left(0.06,0.025^{2},0,0.135\right), & i\in {𝒫}_{2}. \end{array}\right.$$ (1)

Also, we set *I*_ext_ ∼ N(43, 1^2^).

**Figure 5.**
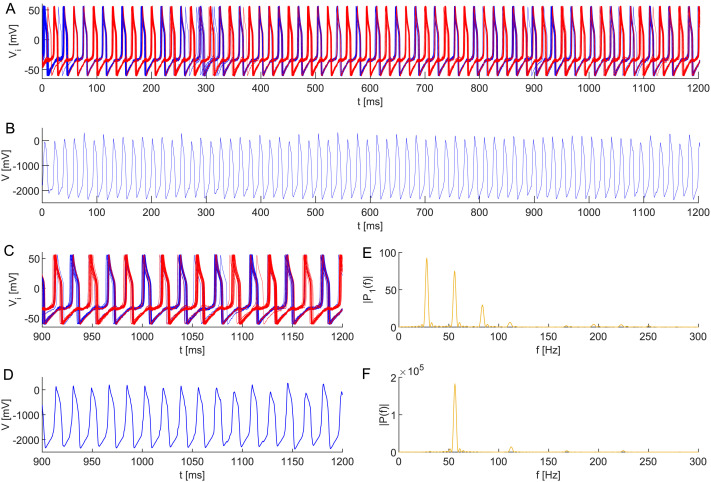
(A) Collective APS of two subpopulations in a neuronal network model (Equation 15) composed of 50 all-to-all coupled Morris–Lecar neurons with heterogeneous membrane capacitances *C*_*i*_ ∼ TN(0.90, 0.05^2^, 0.75, 1.05) and random initial conditions in Equation 1. This setting leads to a doubled dominant frequency in (B) the composed signal *V* = $\sum_{i=1}^{50}$ *V*_*i*_. Zooming in on the dynamics of (C) all neurons, (D) composed signal, and the corresponding periodograms of (E) individual neurons and (F) the composed signal. The robustness of the anti-phase behavior is demonstrated by a noisy applied current *I*_ext_ ∼ N(43, 1^2^) at each simulation step Δ*t* = 0.01. The remaining parameter values are identical (see Table 1), *ε* = 0.002.

Figure 5 shows that this setting leads to anti-phase synchronous oscillations of the two clusters, resulting in a double frequency in the composed signal, as one can see in the periodograms of the clusters and the composition, respectively. Namely, the observed frequency increased to ∼56 Hz. Notice that individual neurons can jump from one cluster to the other due to the noise, while the global behavior remains unchanged.

**Figure 6.**
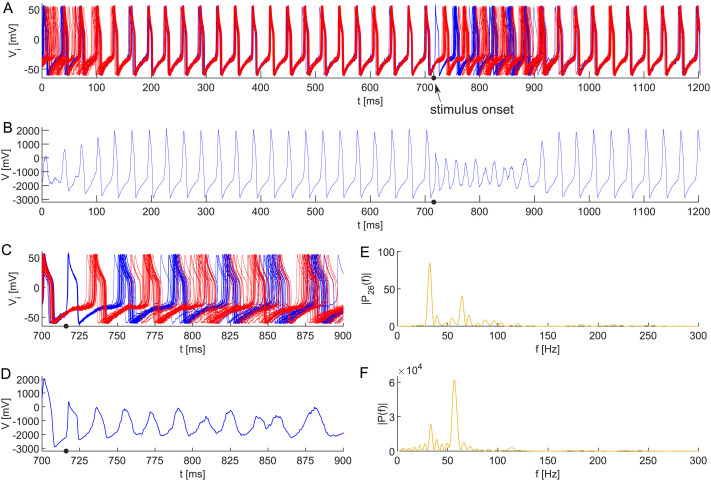
(A) Transient collective APS in a neuronal network model (Equation 15) composed of 50 all-to-all coupled Morris–Lecar neurons with heterogeneous membrane capacitances *C*_*i*_ ∼ TN(1.04, 0.03^2^, 0.95, 1.13) and random initial conditions in Equation 1. The parameter values remain unchanged from the previous setting, with *ε* = 0.002. The APS is evoked by 2 ms lasting additional stimulus at *t* = 716 (arrow). Zooming in on the dynamics of (B) all neurons, (D) composed signal, and the corresponding periodograms of (C) individual neurons and (E) the composed signal.

We acknowledge that the very high-frequency events are only transient phenomena in the experimental observations. We have just shown that with small levels of noise, the anti-phase solution persists. However, higher levels typically lead to complete synchronization (Neiman, Schimansky-Geier, Cornell-Bell, & Moss, 1999; Pikovsky et al., 2001; Zhou & Kurths, 2003). Nevertheless, Figure 6 demonstrates that higher frequencies may temporally appear through external inputs. Let us change the applied current to *I*_ext_ = 43 + *p*_*i*_(*t*) + *σ* d*W* with *σ* = 2.0 and *p*_*i*_ = 40 if *t* ∈ [716, 718] ms in Equation 15 for *i* ∈ 𝒫_1_. This stimulus is applied only to half of the population, that is, *p*_*i*_ = 0 for *i* ∈ 𝒫_2_. We initialize the population into the anti-phase behavior and subsequently observe that the population synchronizes within 100 ms. Looking at the same time interval as before, the external stimulus arrives to halve the population evoking anti-phase behavior that lasts for ∼200 ms; then the population synchronizes again. As the periodograms and time series show, it is evident that the higher frequencies are temporally present. This transient phase solution also appears if we stimulate fewer neurons but then lasts only a few cycles (data not shown). This transient phenomenon depends on the timing of the stimulus as a stimulus during different phases of the period does not induce the anti-phase solution.

### Model Dynamics for Two Coupled Interneurons

The dynamics of two interacting interneurons coupled through gap-junctional connections has already been studied in Chow and Kopell (2000) and Skinner, Zhang, Velazquez, and Carlen (1999). At low coupling strengths and very high firing rates, the synchronous state is unstable, and a pair of cells fires in anti-phase synchrony, while for a lower range of frequencies, the in-phase and anti-phase synchronies may be bistable (Chow & Kopell, 2000). Moreover, Skinner et al. (1999) found that blocking the inhibition in a network of interneurons coupled by gap junctions and inhibitory synapses can lead to anti-phase bursting with higher frequency.

**Figure 7.**
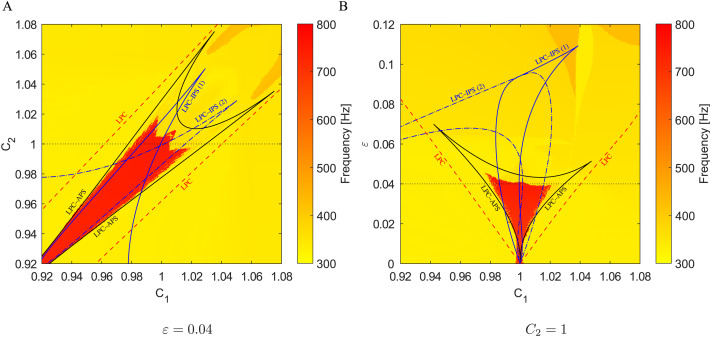
Arnold tongues in parameter spaces (A) *C*_1_ × *C*_2_ and (B) *C*_1_ × *ε* for two coupled interneuron models (Equation 17 and 19) with *I*_ext_ = 24. The regions are delineated by blue and black LPC curves corresponding to the IPS and APS , respectively. The red dashed curves refer to the LPC bifurcation of two unstable periodic solutions. The background color indicates the dominant frequency in a simulated composed signal *V* = *V*_1_ + *V*_2_, reaching up to ∼750 Hz. The yellow region inside the APS tongue delimited by black curves belongs to the signals where the multiple frequency persists, but is not dominant.

**Figure 8.**
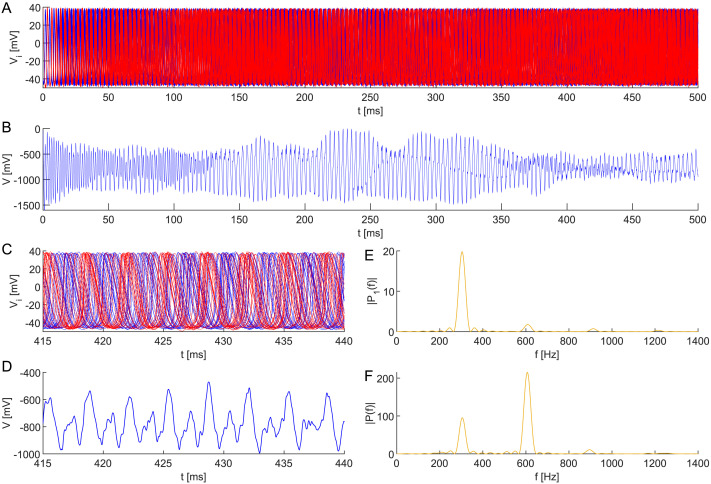
(A) Transient anti-phase dynamics of two subpopulations in a neuronal network model (Equation 17 and 19) composed of 50 all-to-all coupled interneurons with heterogeneous membrane capacitances *C*_*i*_ ∼ TN(1, 0.03^2^, 0.91, 1.09), coupling (Equation 2), and initial conditions (Equation 3). This configuration leads to the rise of VHFOs in (B) the composed signal *V* = $\sum_{i=1}^{50}$ *V*_*i*_. Dynamics of (C) all neurons are zoomed, (D) composed, and depicted with periodograms of (E) individual neurons and (F) the composed signal. The robustness of the anti-phase behavior is demonstrated by a noisy applied direct current *I*_ext_ ∼ N(20, 1^2^) at each simulation step Δ*t* = 0.01. The remaining parameter values are identical.

Similarly, as in the case of Morris–Lecar neuron, let us focus briefly on the dynamics of the interneuron model with respect to parameters *C*_1_, *C*_2_, and *ε*. One can proceed analogously with respect to other parameters affecting the frequency of the membrane potential. Figure 7 shows Arnold tongues in parameter spaces *C*_1_ × *C*_2_ and *C*_1_ × *ε* (*f*–*I* curves for a single interneuron for various values of *C* are provided in the Supporting Information (see Figure S4); the default parameter choice of *I*_ext_ = 24 leads to a frequency of 335 Hz). The blue LPC curves delineate a pair of Arnold tongues corresponding to two symmetrical in-phase solutions; the black tongue refers to the anti-phase synchrony. For the sake of completeness, the red dashed line denotes the LPC curves of two unstable periodic solutions related to the APS. The background color illustrates the dominant frequency detected in a composed signal *V* = *V*_1_ + *V*_2_ simulated on a time interval [500, 1,500], discarding 500 ms transients, for parameter values from a 401 × 401 grid and appropriate initial conditions. These regions do not match precisely, for example, around the point [1, 0] in Figure 7B, that is, for very weak coupling and similar membrane capacitances, where the observed dominant frequency of ∼670 Hz in the composition is caused by transient dynamics (similar to the behavior depicted in Figure 8).

### Simulation of the Transient Collective Anti-Phase Behavior in a Neuronal Network of Coupled Interneurons

Consider the neuronal network (Equation 17 and 19) composed of 50 interacting interneurons formed into two clusters, 𝒫_1_ = {1, …, 25} and 𝒫_2_ = {26, …, 50}, respectively. Further, for the demonstration of the transient anti-phase collective behavior in this system, let the coupling strength take the form

$$\varepsilon_{ij}=\left\{\begin{array}{ll} 0.001, & \left(i,j\right)\in {𝒫}_{1}\times {𝒫}_{1}\cup {𝒫}_{2}\times {𝒫}_{2},\\ 0.0002, & \text{else}, \end{array}\right.$$ (2)

where the first row refers to the coupling within each subpopulation, while the second one indicates a weaker connection between them.

Finally, assume that the clusters start in roughly opposite phases, specifically,

$$V_{i}\left(0\right)=\left\{\begin{array}{ll} 40, & i\in {𝒫}_{1},\\ -40, & i\in {𝒫}_{2}, \end{array}\right.\;h_{i}\left(0\right)=0.25,\;n_{i}\left(0\right)=0.5,\;i\in {𝒫}_{1}\cup {𝒫}_{2}.$$ (3)

Figure 8 reveals the transient anti-phase behavior of these clusters. The periodograms suggest that this dynamics may result in the emergence of VHFOs in the simulation of the composed EEG signal. Namely, summing the signals of individual interneurons resulted in an oscillation with a frequency of ∼610 Hz. Moreover, although transient, the VHFOs may sustain for a relatively long time interval, significantly exceeding the duration of VHFOs observed in real EEG signals (Brázdil et al., 2017; Cimbalnik et al., 2020; Travnicek et al., 2021).

### Transient UFRs and UFOs in a Two-Level Neuronal Network of Coupled Interneurons

**Figure 9.**
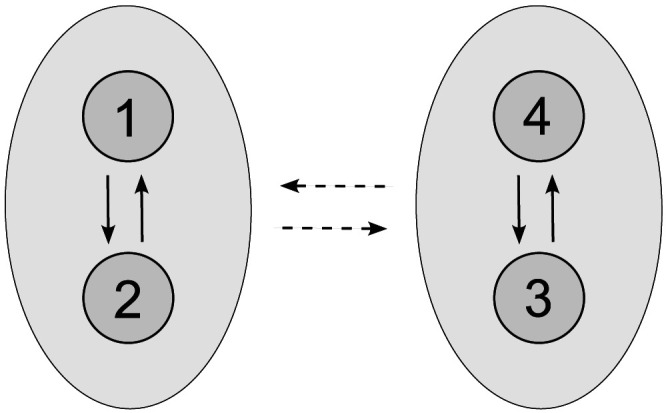
Network topology leading to apparent UFRs; see Figure 10.

Once more, consider the neuronal network (Equation 17 and 19) composed of four interacting interneurons formed into two clusters, 𝒫_1_ = {1, 2} and 𝒫_2_ = {3, 4}, respectively. Suppose that the network connectivity is given by the coupling

$$\varepsilon_{ij}=\left\{\begin{array}{ll} 0.0125, & \left(i,j\right)\in {𝒫}_{1}\times {𝒫}_{1}\cup {𝒫}_{2}\times {𝒫}_{2},\\ 0.0025, & \text{else}; \end{array}\right.$$ (4)

see Figure 9. And finally, assume the following initial conditions:

$$V_{i}\left(0\right)=\left\{\begin{array}{ll} 40, & i\in \left\{1,3\right\},\\ -40, & i\in \left\{2,4\right\}, \end{array}\right.\;h_{i}\left(0\right)=\left\{\begin{array}{ll} 0.25, & i\in {𝒫}_{1},\\ 0.27, & i\in {𝒫}_{2}, \end{array}\right.\;n_{i}\left(0\right)=\left\{\begin{array}{ll} 0.50, & i\in {𝒫}_{1},\\ 0.52, & i\in {𝒫}_{2}. \end{array}\right.$$ (5)

**Figure 10.**
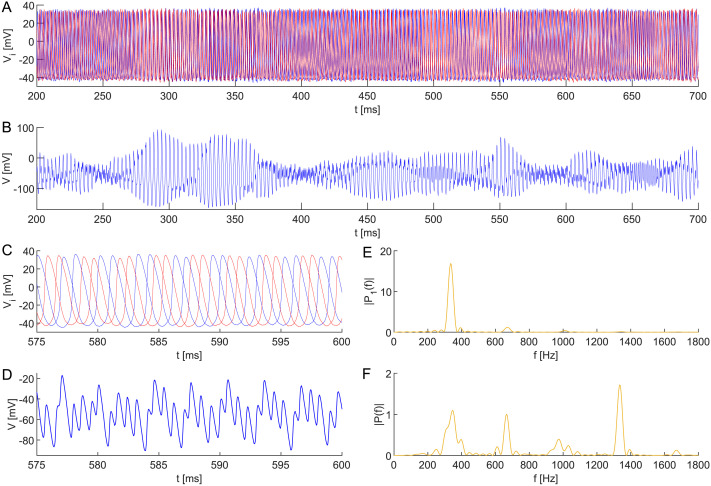
(A) Transient anti-phase dynamics of two subpopulations in a neuronal network model (Equation 17 and 19) composed of four all-to-all coupled interneurons with heterogeneous membrane capacitances *C*_1_ = 0.998, *C*_2_ = 0.999, *C*_3_ = 1, *C*_4_ = 1.001, coupling (Equation 4), and initial conditions (Equation 5). This configuration results in the emergence of UFRs in (B) the composed signal *V* = $\sum_{i=1}^{4}$ *V*_*i*_. Dynamics of (C) all neurons are zoomed, (D) composed, and depicted with periodograms of (E) individual neurons and (F) the composed signal. The robustness of the anti-phase behavior is demonstrated by a noisy applied direct current *I*_ext_ ∼ N(24, 1^2^) at each simulation step Δ*t* = 0.01. The remaining parameter values are identical.

As one can see in Figure 10, this configuration leads to the emergence of transient UFRs in the composed signal *V* = $\sum_{i=1}^{4}$ *V*_*i*_. Specifically, in spite of the noisy applied direct current, the signal simulation contains a segment reporting a frequency of ∼1335 Hz. Despite being transient, the simulated UFRs exhibit a prolonged duration compared to the UFRs and UFOs observed in actual EEG records (Brazdil et al., 2023; Travnicek et al., 2021).

**Figure 11.**
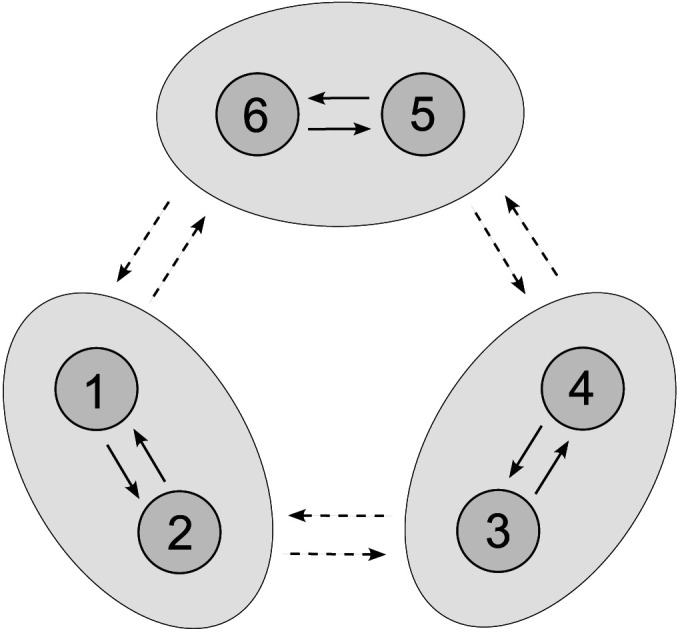
Network topology leading to apparent UFOs; see Figure 12.

Moreover, let us extend the previous neuronal network model by a third pair of interneurons. Specifically, consider six interacting interneurons organized into three clusters, 𝒫_1_ = {1, 2}, 𝒫_2_ = {3, 4}, and 𝒫_3_ = {5, 6}, respectively; assume that the network connectivity is determined by the coupling

$$\varepsilon_{ij}=\left\{\begin{array}{ll} 0.01, & \left(i,j\right)\in {𝒫}_{1}\times {𝒫}_{1}\cup {𝒫}_{2}\times {𝒫}_{2}\cup {𝒫}_{3}\times {𝒫}_{3},\\ 0.001, & \text{else}; \end{array}\right.$$ (6)

see Figure 11. Finally, let the initial state of the system satisfy

$$V_{i}\left(0\right)=\left\{\begin{array}{ll} 40, & i\in \left\{1,3,5\right\},\\ -40, & i\in \left\{2,4,6\right\}, \end{array}\right.\;h_{i}\left(0\right)=\left\{\begin{array}{ll} 0.25, & i\in {𝒫}_{1},\\ 0.26, & i\in {𝒫}_{2},\\ 0.27, & i\in {𝒫}_{3}, \end{array}\right.\;n_{i}\left(0\right)=\left\{\begin{array}{ll} 0.50, & i\in {𝒫}_{1},\\ 0.51, & i\in {𝒫}_{2},\\ 0.52, & i\in {𝒫}_{3}, \end{array}\right.$$ (7)

characterizing anti-phase synchrony within each cluster and slightly shifted phases between clusters.

**Figure 12.**
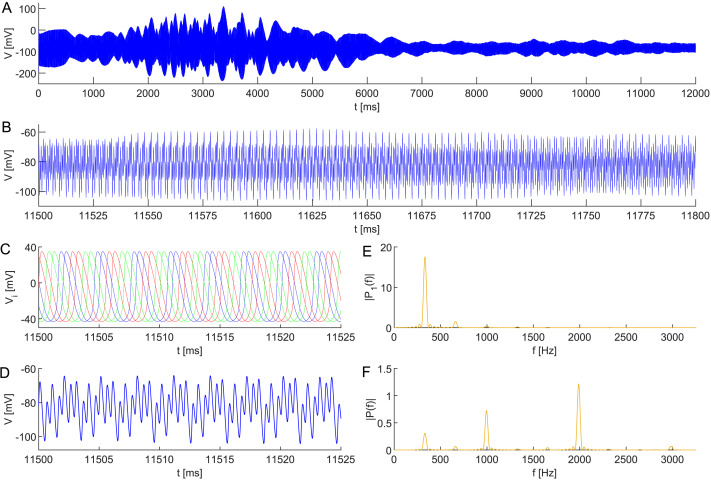
Stable dynamics of three subpopulations in a neuronal network model (Equation 17 and 19) comprising six all-to-all coupled interneurons with heterogeneous membrane capacitances *C*_*i*_ ∼ TN(1, 0.001^2^, 0.997, 1.003), coupling (Equation 6), and initial conditions (Equation 7). This configuration results in the birth of UFOs in (A), (B) the composed signal *V* = $\sum_{i=1}^{6}$ *V*_*i*_. Dynamics of (C) all neurons are shown in detail, (D) composed, and depicted with periodograms of (E) individual neurons and (F) the composed signal. The robustness of the regular shift-phase behavior is demonstrated by a noisy applied direct current *I*_ext_ ∼ N(24, 0.03^2^) at each simulation step Δ*t* = 0.01. The remaining parameter values are identical.

As Figure 12 shows, this setting results in the emergence of UFOs in the composed signal *V* = $\sum_{i=1}^{6}$ *V*_*i*_. Namely, despite the noisy applied direct current, after a transient period, the simulated signal composition oscillates with a frequency of ∼2 kHz.

### Simulation of the Collective Anti-Phase Behavior in a Model of Destexhe–Paré Neuronal Network

Let us take into account the neuronal network (Equation 20 and 21) comprised of 50 coupled Destexhe–Paré neurons formed into two clusters, 𝒫_1_ = {1, …, 25} and 𝒫_2_ = {26, …, 50}, respectively. Unlike previous cases, here we assume that the distribution of capacitances differs in the mean value between these two groups. Namely, let

$$C_{i}∼\text{TN}\left(0.95,0.03^{2},0.86,1.04\right),\;i\in {𝒫}_{1},\;\text{and}\;C_{i}∼\text{TN}\left(1,0.03^{2},0.91,1.09\right),\;i\in {𝒫}_{2}.$$ (8)

Let the network connectivity take the form

$$\varepsilon_{ij}=\left\{\begin{array}{ll} 0.005, & \left(i,j\right)\in {𝒫}_{1}\times {𝒫}_{1}\cup {𝒫}_{2}\times {𝒫}_{2},\\ 0.001, & \text{else}, \end{array}\right.$$ (9)

characterizing the coupling strength within each subpopulation and between them, respectively.

And finally, for the demonstration of transient anti-phase behavior in this network, suppose that the clusters start in roughly opposite phases; specifically, let

$$V_{i}\left(0\right)=\left\{\begin{array}{ll} -75, & i\in {𝒫}_{1},\\ 35, & i\in {𝒫}_{2}, \end{array}\right.\;m_{i}\left(0\right)=0.5,\;h_{i}\left(0\right)=0.2,\;n_{i}\left(0\right)=0.4,\;m_{\text{M},i}\left(0\right)=0.24,\;i\in {𝒫}_{1}\cup {𝒫}_{2}.$$ (10)

**Figure 13.**
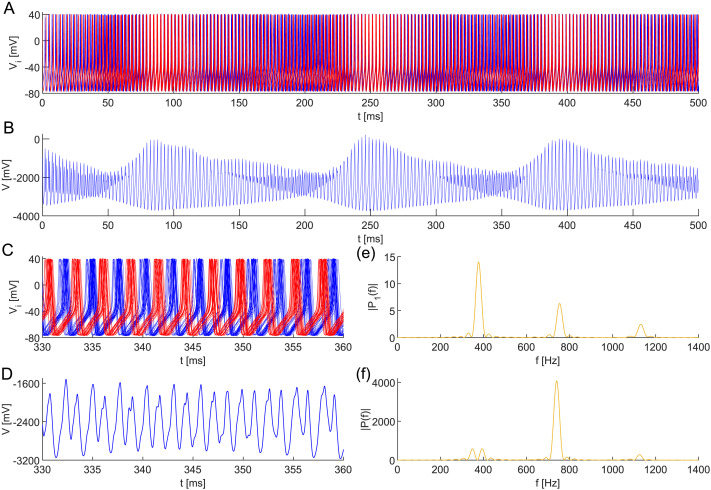
(A) Transient anti-phase dynamics of subpopulations 𝒫_1_, 𝒫_2_ in a neuronal network model Equation 20 and 21 composed of 50 all-to-all coupled Destexhe–Paré neurons with heterogeneous membrane capacitances (Equation 8), coupling (Equation 9), and initial conditions (Equation 10). Dynamics of (B) composed signal, zoomed dynamics of (C) individual neurons, and (D) composed signal are depicted with periodograms of (E) individual neurons and (F) the composed signal. The robustness of the anti-phase behavior is demonstrated by a noisy applied direct current *I*_ext_ ∼ N(40, 1^2^) at each simulation step Δ*t* = 0.01. The remaining parameter values are identical.

The transient anti-phase behavior of these clusters is presented in Figure 13. At the bottom, one can see signal segments with the examined dynamics. The periodograms corresponding to these segments demonstrate that the VHFOs are present in the composed EEG signal simulation. To be more specific, the combination of signals from each neuron leads to an oscillation with a frequency of ∼740 Hz (*f*–*I* curves for a single neuron for various values of *C* are provided in the Supporting Information (see Figure S5); the default parameter choice of *I*_ext_ = 40 leads to a frequency of 360 Hz). Although transient, the VHFOs can persist for a relatively long period, significantly surpassing the length of VHFOs detected in actual EEG signals (Brázdil et al., 2017; Brazdil et al., 2023; Cimbalnik et al., 2020; Travnicek et al., 2021).

## DISCUSSION AND CONCLUSIONS

The primary objective of our paper was to establish a rigorous basis for the possibility that a reduction in the strength of the connections between neurons in an epileptic focus could produce high, very high-frequency, and ultra-fast oscillations measured at microelectrodes in its vicinity. Although such oscillations are biomarkers used for diagnostic purposes in presurgical evaluation, there is still no definitive way to differentiate between physiological (most commonly associated with the normal physiological function of cognitive or sensory processing (see Pail et al., 2020) and pathological HFOs in EEG signals (Frauscher et al., 2018). There are recent studies (Cimbalnik et al., 2018, 2020; Nejedly et al., 2019) that introduced algorithms for the detection of pathological HFOs using pathological events visually marked by expert reviewers inside the seizure onset zone of patients with focal epilepsy (detection training and machine learning), but finding a way to distinguish between physiological and pathological HFOs remains challenging. In contrast, VHFOs and UFOs are likely to be important markers of epileptogenicity (Brázdil et al., 2017), and a plausible mechanistic explanation of such high frequencies in the LFP signal adds to the understanding of the pathophysiology and improving the treatment of drug-resistant focal epilepsy or the development of new less invasive tools for its treatment.

We established that synchronization of a weakly coupled neuronal network occurs in a large parameter region. The phenomenon of multistability allows for the synchronization of in-phase, anti-phase, or other spatiotemporally symmetric phase shifts. This enables the network to generate tonic spiking at its own frequency and simultaneously produce spikes at a regularly shifted phase. This phenomenon potentially creates an event that can be recorded as a very high-frequency or ultra-fast oscillation by nearby electrodes. One assumes the recorded signal is a weighted sum of the nearby activity (see Katzner et al., 2009; Mitzdorf, 1985), but here, for simplicity, all neurons contribute equally as this choice does not affect the dominant frequency. We simulated the network for different parameters in physiological ranges and subjected the system to an external current with random noise around a reference value. We found that synchronized VHFOs and UFOs are possible for very weakly coupled nearly identical neurons, persisting even with small random noise affecting the magnitude of the external current input. Our findings resonate with prior work highlighting already fast ripples as a network phenomenon in epileptic rodents, driven by out-of-phase synchronized firing across neuron pools (see Jefferys et al., 2012).

In this study, we implemented gap-junctional coupling into our model because the reactions of the dynamics would manifest more slowly if chemical synapses were utilized, particularly in the context of modeling very high-frequency oscillations. Gap junctions, as electrical synapses, are known for their ability to support rapid and bidirectional communication between neurons, which makes them suitable for modeling high-frequency oscillatory dynamics. However, we acknowledge the intricate, multifaceted nature of neuronal networks and therefore concede that both gap junctions and chemical synapses might play significant roles in the overall dynamic behavior. Nevertheless, we have shown that the phenomenon of frequency multiplication is generic and model-independent. There are other, more complex models with gap-junctional coupling but random (Erdös–Renyi graph) connectivity that could show the same (see Ghosh et al., 2020). Initial simulations showed emergent network oscillations with higher frequencies (up to 70 Hz instead of the individual 10 Hz) as the coupling strength increased. We observed that the network fired in clusters, but neurons did not always participate in the active cluster. So, while our mechanism seems relevant, a complete characterization requires a separate study. Future studies might benefit from models that concurrently integrate both types of synapses to capture a broader range of neuronal dynamics and interactions.

We used gap-junctional coupling of near-identical neurons of the same type for our analysis, but other parameters can also be varied with similar results. Specifically, we have already examined rigorously that slight changes in the sodium conductance *g*_Na_ and reversal potential *V*_Na_ in the interneuron model do not affect the obtained results. This will be reported elsewhere. Moreover, we found that excitation with a noisy external current gives analogous results. The approach is applicable to a multilevel network comprising subgroups of neurons that already exhibit VHFOs through anti-phase spiking within each subgroup. As a result, the presence of weak coupling between these subgroups gives rise to transient UFRs and UFOs in the simulated EEG signal. However, there is still much to be explored, including whether VHFOs and UFOs can be simulated in the EEG signal for a more complex network composed of multiple different types of neurons and how the network connectivity matrix affects the dynamics. Moreover, the persisting stability of anti-phase oscillations in a multilevel network is currently undetermined, as their stability and resilience have been established in single-level and two-level networks with very weak coupling. However, it is likely that the stability region will diminish in the multilevel scenario.

Our results support the hypothesis of a spatially limited pathological phenomenon, which is also manifested in VHFOs and UFOs. In addition, the coupling type and its strength can affect the dynamics of the coupled neurons, with strong enough coupling leading to in-phase synchrony or small phase-shift synchrony and very weak coupling allowing stable anti-phase regime, that may, in that case, temporally appear in the EEG signal. This result is consistent with the observation of altered connectivity in epileptic brain networks (see DeSalvo, Douw, Tanaka, Reinsberger, & Stufflebeam, 2014).

In conclusion, our study provides a mathematical framework for investigating the occurrence of HFOs, VHFOs, and UFOs in the EEG signals of patients with focal epilepsy. Our findings suggest that a reduction in the strength of the connections between neurons in an epileptic focus can produce very high-frequency signals, which could aid in understanding the pathology and improving the treatment of focal epilepsy. However, there is still much to be explored regarding the coupling types, their strength, and other parameters that could affect the dynamics of the coupled neurons.

## METHODS

### Morris–Lecar Neuron Model

The Morris–Lecar model was developed by Morris and Lecar (1981) to reproduce the variety of oscillatory behavior in relation to Ca^2+^ and K^+^ conductance (van Putten, 2020). Although it was originally created to model the potential in the muscle fiber of the giant barnacle, the Morris–Lecar model is today used for modeling brain pyramidal neurons due to its simplicity and ability to model various dynamics (Lecar, 2007; Prescott, De Koninck, & Sejnowski, 2008; Prescott, Ratté, et al., 2008). The model is described by the following two-dimensional system of differential equations

$$\begin{array}{l} C\frac{\text{d}}{\text{d}t}V=I_{\text{ext}}-g_{\text{L}}\left(V-V_{\text{L}}\right)-g_{\text{Ca}}m_{\infty }\left(V\right)\left(V-V_{\text{Ca}}\right)-g_{\text{K}}w\left(V-V_{\text{K}}\right),\\ \;\frac{\text{d}}{\text{d}t}w=\frac{w_{\infty }\left(V\right)-w}{\tau_{w}\left(V\right)}, \end{array}$$ (11)

where *V* is the membrane potential [mV] and *w* represents the activation variable for K^+^, that is, the probability that the K^+^ channel is conducting. The parameter *C* corresponds to the cell membrane capacitance.

In comparison to the K^+^ current, the Ca^2+^ current changes rapidly, and its activation is assumed to be instantaneous. The activation function is modeled as

$$m_{\infty }\left(V\right)=\frac{1}{2}\left[1+\tanh\left(\frac{V-\beta_{1}}{\beta_{2}}\right)\right],$$ (12)

where *β*_1_ and *β*_2_ denote the potential at which the Ca^2+^ current is half-activated and the slope of the activation voltage dependence, respectively.

Similarly, for the voltage-dependent steady state *w*_∞_, we use the form

$$w_{\infty }\left(V\right)=\frac{1}{2}\left[1+\tanh\left(\frac{V-\beta_{3}}{\beta_{4}}\right)\right],$$ (13)

where *β*_3_ and *β*_4_ have analogous meanings to *β*_1_ and *β*_2_, respectively. The timescale of the *w* variable is given by

$$\tau_{w}\left(V\right)=\frac{1}{2}{\left[\varphi \cosh\left(\frac{V-\beta_{3}}{2\beta_{4}}\right)\right]}^{-1},$$ (14)

where the parameter *φ* is a temperature factor adjusting the relative timescale of *V* and *w*.

The system parameters *g*_L_, *g*_Ca_, and *g*_K_ represent the maximum leak, Ca^2+^, and K^+^ electrical conductances through membrane channels, respectively, and *V*_L_, *V*_Ca_, and *V*_K_ are reversal potentials of the specific ion channels. Finally, *I*_ext_ denotes the externally applied direct current, whichdirect can be used as a single parameter determining a class of the studied cell, that is, whether we deal with class I or class II neuron according to the classification proposed by Hodgkin (1948).

Unless otherwise stated, we make the parameter choices as listed in Table 1. These physiological parameter values were taken from Gutkin and Ermentrout (1998). Characteristic values for the hippocampal cell membrane capacitance *C* are found to be around 1 *μ*F/cm^2^ (Destexhe & Paré, 1999; Ermentrout & Terman, 2010; Hartveit, Veruki, & Zandt, 2019; Keener & Sneyd, 2009; Tewari et al., 2018). Therefore, within the framework of this article, we consider these values.

### Model of Morris–Lecar Neuronal Network

Timescales of synapses of neurons and gap junctions differ. Specifically, synaptic coupling refers to the mechanism whereby neurotransmitters are released from a presynaptic neuron and subsequently bind to receptors located on a postsynaptic neuron, inducing changes in the latter’s membrane potential. This process is relatively slow, taking several milliseconds to occur. In contrast, gap junctions provide direct electrical communication between neurons, allowing for very rapid transmission of electrical signals with virtually no delay in the communication between the coupled neurons. Given our objective to model high frequencies, we opted for a network model that incorporates gap junctions, which are known to be present in the mammalian central nervous system, including the hippocampus (Draguhn, Traub, Schmitz, & Jefferys, 1998; Fukuda & Kosaka, 2000). Morris–Lecar neuronal networks with gap junctions have recently been studied with regard to epilepsy (Volman, Perc, & Bazhenov, 2011; Naze, Bernard, & Jirsa, 2015). HFOs have also been reported in a computational study with Hodgkin–Huxley type models with gap junctions by Helling, Koppert, Visser, and Kalitzin (2015). Here, we examine a small population of neurons with gap junction coupling as it has been found that the epileptic brain tissue may be functionally isolated from surrounding brain regions (see Klimes et al., 2016; Warren et al., 2010). Moreover, the review of Jin and Zhong (2011) focuses on gap junctions in the pathophysiology of epilepsy, describing their role in the generation, synchronization, and maintenance of seizures, and points to gap junctions as promising targets for the development of antiseizure medication, emphasizing the need for further research in this field to refine our understanding and treatment of epilepsy.

Let us examine a neuronal network model of *N* electrically coupled Morris–Lecar neurons (Equation 11) described by

$$\begin{array}{l} C_{i}\frac{\text{d}}{\text{d}t}V_{i}=I_{\text{ext}}-g_{\text{L}}\left(V_{i}-V_{\text{L}}\right)-g_{\text{Ca}}m_{\infty }\left(V_{i}\right)\left(V_{i}-V_{\text{Ca}}\right)-g_{\text{K}}w_{i}\left(V_{i}-V_{\text{K}}\right)-\sum_{j=1}^{N}\varepsilon_{ij}\left(V_{i}-V_{j}\right),\\ \;\frac{\text{d}}{\text{d}t}w_{i}=\frac{w_{\infty }\left(V_{i}\right)-w_{i}}{\tau_{w}\left(V_{i}\right)}, \end{array}$$ (15)

where *i* ∈ {1, …, *N*} and *K* = ${\left(\varepsilon_{ij}\right)}_{i,j=1}^{N}$ represents a coupling matrix determining the network topology, that is, its connectivity. Within this article, concerning the Morris–Lecar model, we focus on the case of an all-to-all coupled neuronal network with identical coupling strength

$$\varepsilon_{ij}=\varepsilon =\frac{0.1}{N}.$$ (16)

The functions *m*_∞_(*V*), *w*_∞_(*V*), and *τ*_*w*_(*V*) are defined in Equation 12, 13, and 14, respectively. Initial conditions for gating variable: *w*_1_(0) = *w*_2_(0) = 0.04 unless specified otherwise.

### Interneuron Model

Consider a neuronal network composed of *N* electrically coupled interneurons (White, Chow, Rit, Soto-Treviño, & Kopell, 1998). Let the membrane potential *V*_*i*_ [mV] of neuron *i* satisfy the equation

$$C_{i}\frac{\text{d}}{\text{d}t}V_{i}=I_{\text{ext}}-I_{\text{L}}-I_{\text{Na}}-I_{\text{K}}-\sum_{j=1}^{N}\varepsilon_{ij}\left(V_{i}-V_{j}\right),$$ (17)

where *C*_*i*_ represents the membrane capacitance, *I*_ext_ is the externally applied current, and *I*_L_, *I*_Na_, and *I*_K_ stand for the Hodgkin–Huxley type leak, Na^+^, and K^+^ currents, respectively. The last term represents the gap-junctional coupling between neuron *i* and all other neurons in the network, where *ε*_*ij*_ is the gap junction conductance. The Na^+^ and K^+^ currents are given by

$$I_{\text{Na}}=g_{\text{Na}}m_{i}^{3}h_{i}\left(V_{i}-V_{\text{Na}}\right)\;\text{and}\;I_{\text{K}}=g_{\text{K}}n_{i}^{4}\left(V_{i}-V_{\text{K}}\right),$$ (18)

where *g*_Na_ and *g*_K_ are the maximum conductances, *m*_*i*_ and *h*_*i*_ represent gating variables for Na^+^ channels, and *n*_*i*_ is a gating variable for K^+^ channels. The leak current is given by *I*_L_ = *g*_L_(*V*_*i*_ − *V*_L_), where *g*_L_ stands for the leak conductance and *V*_L_ is the leak reversal potential.

The activation variable *m*_*i*_ is assumed to tend rapidly to its steady state. Hence one can substitute it by its asymptotic value *m*_*i*_ = *m*_∞_(*V*_*i*_) = (1 + exp [−0.08(*V*_*i*_ + 26)])^−1^. Further, the gating variables *h*_*i*_ and *n*_*i*_ obey

$$\frac{\text{d}}{\text{d}t}h_{i}=\frac{h_{\infty }\left(V_{i}\right)-h_{i}}{\tau_{h}\left(V_{i}\right)}\;\text{and}\;\frac{\text{d}}{\text{d}t}n_{i}=\frac{n_{\infty }\left(V_{i}\right)-n_{i}}{\tau_{n}\left(V_{i}\right)}$$ (19)

with

$$h_{\infty }\left(V\right)=\frac{1}{1+\exp\left[0.13\left(V+38\right)\right]},\;\tau_{h}\left(V\right)=\frac{0.6}{1+\exp\left[-0.12\left(V+67\right)\right]},$$

$$n_{\infty }\left(V\right)=\frac{1}{1+\exp\left[-0.045\left(V+10\right)\right]},\;\tau_{n}\left(V\right)=0.5+\frac{2}{1+\exp\left[0.045\left(V-50\right)\right]},$$

respectively.

In this article, we consider parameter values *C* ≈ 1 *μ*S/cm^2^, *g*_L_ = 0.1 mS/cm^2^, *g*_Na_ = 30 mS/cm^2^, *g*_K_ = 20 mS/cm^2^, *V*_L_ = −60 mV, *V*_Na_ = 45 mV, and *V*_K_ = −80 mV. These values are within physiological ranges and give the high-frequency firing rates typical of hippocampal interneurons (White et al., 1998).

### Destexhe–Paré Neuron Model

The last model of a hippocampal pyramidal cell we consider within this paper comes from Destexhe and Paré (1999). Currents dynamics are described by Hodgkin–Huxley type models with kinetics based on a model of hippocampal pyramidal cells from Traub and Miles (1991), adjusted to match voltage-clamp data of cortical pyramidal cells (Huguenard, Hamill, & Prince, 1988), and a noninactivating K^+^ current was described in Mainen, Joerges, Huguenard, and Sejnowski (1995).

Once again, we examine a neuronal network comprising *N* coupled neurons. Let the membrane potential *V*_*i*_ [mV] of neuron *i* be described by equation

$$C_{i}\frac{\text{d}}{\text{d}t}V_{i}=I_{\text{ext}}-I_{\text{L}}-I_{\text{Na}}-I_{\text{Kdr}}-I_{\text{M}}-\sum_{j=1}^{N}\varepsilon_{ij}\left(V_{i}-V_{j}\right),$$ (20)

where *C*_*i*_ denotes the membrane capacitance; *I*_ext_ is the externally applied direct current, and *I*_L_, *I*_Na_, *I*_Kdr_, and *I*_M_ represent the leak, Na^+^, and K^+^ currents. The sum term stands for the gap junction coupling between neuron *i* and all other neurons in the network with the gap junction conductance *ε*_*ij*_. As usual, the leak current is given by *I*_L_ = *g*_L_(*V*_*i*_ − *V*_L_), where *g*_L_ is the leak conductance, and *V*_L_ denotes the leak reversal potential. As mentioned, all gating variables *p* ∈ {*m*, *h*, *n*, *m*_M_} obey first-order kinetics

$$\frac{\text{d}}{\text{d}t}p_{i}=\alpha_{p}\left(V_{i}\right)\left(1-p_{i}\right)-\beta_{p}\left(V_{i}\right)p_{i}$$ (21)

with functions *α*_*p*_(*V*_*i*_) and *β*_*p*_(*V*_*i*_) taking the form specific for each variable.

The voltage-dependent Na^+^ current was described by Traub and Miles (1991) and is given by

$$I_{\text{Na}}=g_{\text{Na}}m_{i}^{3}h_{i}\left(V_{i}-V_{\text{Na}}\right),$$ (22)

where *g*_Na_ stands for its maximum conductance, *V*_Na_ is its reversal potential, and *m*_*i*_ and *h*_*i*_ represent gating variables satisfying Equation 21 with

$$\alpha_{m}\left(V\right)=\frac{-0.32\left(V-V_{\text{T}}-13\right)}{\exp\left[-\left(V-V_{\text{T}}-13\right)/4\right]-1},\;\beta_{m}\left(V\right)=\frac{0.28\left(V-V_{\text{T}}-40\right)}{\exp\left[\left(V-V_{\text{T}}-40\right)/5\right]-1},$$

$$\alpha_{h}\left(V\right)=0.128\exp\left[-\left(V-V_{\text{T}}-V_{\text{S}}-17\right)/18\right],\;\beta_{h}\left(V\right)=\frac{4}{1+\exp\left[-\left(V-V_{\text{T}}-V_{\text{S}}-40\right)/5\right]}$$

for *V*_T_ = −58 mV and *V*_S_ = −10 mV fitting the voltage-clamp data.

The current corresponding to the delayed rectifier K^+^ channel was studied by Traub and Miles (1991) and is given by

$$I_{\text{Kdr}}=g_{\text{Kdr}}n_{i}^{4}\left(V_{i}-V_{\text{K}}\right),$$ (23)

where *g*_Kdr_ is the maximum conductance, *V*_K_ represents its reversal potential, and *n*_*i*_ obeys Equation 21 with

$$\alpha_{n}\left(V\right)=\frac{-0.032\left(V-V_{\text{T}}-15\right)}{\exp\left[-\left(V-V_{\text{T}}-15\right)/5\right]-1},\;\beta_{n}\left(V\right)=0.5\exp\left[-\left(V-V_{\text{T}}-10\right)/40\right].$$

And finally, the noninactivating current was described in Mainen et al. (1995) and takes the form

$$I_{\text{M}}=g_{\text{M}}m_{\text{M},i}\left(V_{i}-V_{\text{K}}\right),$$ (24)

where *g*_M_ denotes the maximum conductance and *m*_M_ obeys Equation 21 with

$$\alpha_{m_{\text{M}}}\left(V\right)=\frac{0.0001\left(V+30\right)}{1-\exp\left[-\left(V+30\right)/9\right]},\;\beta_{m_{\text{M}}}\left(V\right)=\frac{-0.0001\left(V+30\right)}{1-\exp\left[\left(V+30\right)/9\right]}.$$

Within this paper, we consider the following physiological parameter values for a hippocampal pyramidal cell taken from Destexhe and Paré (1999). Specifically, *C* ≈ 1 *μ*S/cm^2^, *g*_L_ = 0.019 mS/cm^2^, *g*_Na_ = 120 mS/cm^2^, *g*_Kdr_ = 100 mS/cm^2^, *g*_M_ = 2 mS/cm^2^, *V*_L_ = −65 mV, *V*_Na_ = 55 mV, and *V*_K_ = − 85 mV.

## NUMERICAL METHODS

We used the standard ode45 solver in matlab for deterministic settings, while for simulations with noisy input, we add noise to the external current at each simulation step with Δ*t* = 0.01 using the Euler–Maruyama method. Bifurcation curves have been computed using the numerical bifurcation package MatCont (Dhooge, Govaerts, & Kuznetsov, 2003; Dhooge, Govaerts, Kuznetsov, Meijer, & Sautois, 2008).

## ACKNOWLEDGMENTS

The authors thank Pavel Jurák and Jan Cimbálník for their insightful comments and critical reading of the work before submission.

## SUPPORTING INFORMATION

Supporting information for this article is available at https://doi.org/10.1162/netn_a_00351. Code is available at https://gitlab.ics.muni.cz/9607/UFOs.

## AUTHOR CONTRIBUTIONS

Lenka Přibylová: Conceptualization; Formal analysis; Funding acquisition; Methodology; Project administration; Supervision; Validation; Writing – original draft. Jan Ševčík: Data curation; Formal analysis; Methodology; Software; Visualization; Writing – original draft. Veronika Eclerová: Conceptualization; Methodology; Validation; Writing – review & editing. Petr Klimeš: Validation; Writing – review & editing. Milan Brázdil: Supervision; Validation; Writing – review & editing. Hil Meijer: Methodology; Supervision; Validation; Writing – review & editing.

## FUNDING INFORMATION

Lenka Přibylová, Masarykova Univerzita (https://dx.doi.org/10.13039/501100010653), Award ID: MUNI/G/1213/2022. Jan Ševčík, Masarykova Univerzita (https://dx.doi.org/10.13039/501100010653), Award ID: MUNI/G/1213/2022. Veronika Eclerová, Masarykova Univerzita (https://dx.doi.org/10.13039/501100010653), Award ID: MUNI/G/1213/2022. Milan Brázdil, Masarykova Univerzita (https://dx.doi.org/10.13039/501100010653), Award ID: MUNI/G/1213/2022, Grantová Agentura České Republiky (https://dx.doi.org/10.13039/501100001824), Award ID: GA22-28784S, and European Union – Next Generation EU, Award ID: LX22NPO5107 (MEYS). Petr Klimeš, Grantová Agentura České Republiky (https://dx.doi.org/10.13039/501100001824), Award ID: GA22-28784S, and European Union – Next Generation EU, Award ID: LX22NPO5107 (MEYS).

## Supplementary Material



## TECHNICAL TERMS

Hopf bifurcation (H): A change in a dynamical system that is related to cycle birth from equilibrium.
Limit point of cycles (LPC): A point in a dynamical system representing the fold of a sequence of periodic orbits or cycles as a parameter changes.
Phase-locking: A state in a dynamical system where oscillators synchronize their frequencies, maintaining a constant relative phase difference over time.
Local field potentials: Electrophysiological signals reflecting the summed electrical activity of a group of neurons in a local area.
Rigid: In the context of dynamical systems, refers to behaviors that do not vary or change under a set of conditions.
Phase-shift synchrony: A synchronous state where oscillating systems align their cycles but with a constant time delay or phase difference.
Saddle-node on an invariant curve (SNIC) bifurcation: A critical event in a dynamical system where a stable limit cycle is created or destroyed alongside an equilibrium point.
Saddle-node equilibrium (LP): A state in a dynamical system where two equilibrium points, a stable and an unstable one, merge and annihilate each other.
Subcritical Hopf bifurcation: A system transition where an unstable limit cycle exists before the bifurcation point and a stable equilibrium point loses stability after it.
Bifurcation: A critical point in a dynamical system where a small change in system parameter values results in a qualitative change in its behavior.
Two-dimensional invariant torus: A set of solutions in a dynamical system that form a toroidal shape, wherein trajectories neither converge nor diverge but remain on the torus.
Quasi-periodic: A behavior in a dynamical system with oscillations at two or more incommensurable (irrational ratio) frequencies, creating a nonrepeating pattern.
Period-doubling bifurcation (PD): A transition in a dynamical system where a small change in parameters causes the period of oscillations to double, often preceding chaos.
Arnold tongues: Regions in parameter space indicating synchronization or frequency-locking ranges in dynamical systems, typically with a characteristic ’tongue’ shape.
Synaptic coupling: The process where activity in one neuron influences another through synaptic transmission, facilitating communication and synchronization between neurons.
Gap junction: A specialized intercellular connection that allows direct electrical and chemical communication between neighboring cells, facilitating synchronization of activity.
