# Supplementary material for: Weak coupling of neurons enables very high-frequency and ultra-fast oscillations through the interplay of synchronized phase shifts
Source: Netw Neurosci. 2024 Apr 1;8(1):293–318. doi: 10.1162/netn_a_00351 (PMC10954350; doi:10.1162/netn_a_00351)
Supplement: Supplementary file 1 [file netn-8-1-293-s001.pdf]

Journal: **NETWORK NEUROSCIENCE**

APPENDIX

Weak coupling of neurons enables very high-frequency and ultra-fast oscillations through the interplay of synchronized phase-shifts

Lenka Přibylová[1], Jan Ševčík[1], Veronika Eclerová[1], Petr Klimeš[2], Milan Brázdil[3,4,#], and Hil Meijer[5,#]

[1] Department of Mathematics and Statistics, Faculty of Science, Masaryk University, Brno, Czech Republic

[2] Institute of Scientific Instruments, The Czech Academy of Sciences, Brno, Czech Republic

[3] Brno Epilepsy Center, Dept. of Neurology, St. Anne's Univ. Hospital and Faculty of Medicine, Masaryk University, Brno, Czech Republic, member of the ERN EpiCARE

[4] Behavioral and Social Neuroscience Research Group, Central European Institute of Technology, Masaryk University, Brno, Czech Republic

[5] Department of Applied Mathematics, Techmed Centre, University of Twente, Enschede, The Netherlands

[#] senior supervisor, the last author

Simulations of Morris two bidirectionally coupled Morris–Lecar neurons for $C_1 = 1.2$, $C_2 = 1$ that synchronize in-phase (Fig. S1) with their natural frequency $\sim 30\,\mathrm{Hz}$ emphasized by the dashed line in the Fig. 3 of the main article. The summed signal exhibits the same frequency.

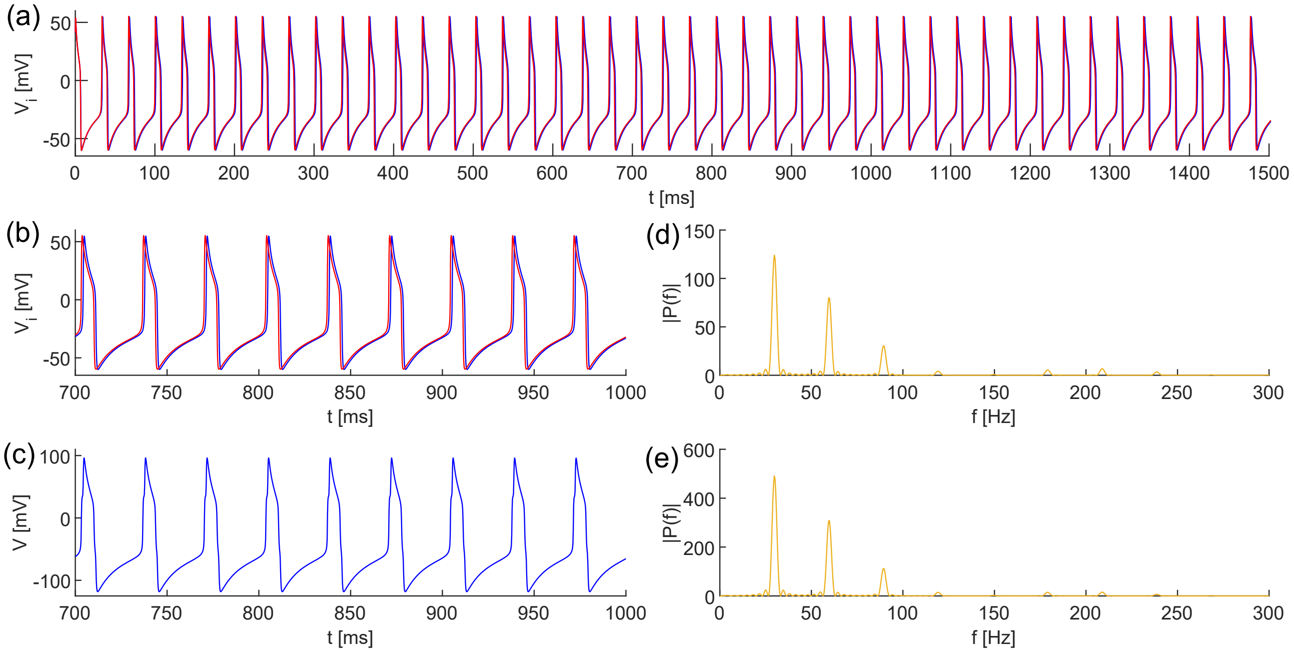

Fig. S1: (a) IPS of two bidirectionally coupled Morris–Lecar neurons for $C_1 = 1.2$, $C_2 = 1$, and initial conditions $V_1(0) = V_2(0) = 40$, $w_1(0) = w_2(0) = 0.04$ with a close-up of the dynamics of (b) V_1 and V_2, (c) $V_1 + V_2$. The remaining parameter values are listed in Table 1 of the main article, $\varepsilon = 0.05$. The periodograms corresponding to (d) individual neurons and (e) the composed signal $V = V_1 + V_2$, both with a dominant frequency of $\sim 30\,\mathrm{Hz}$, indicate that this state does not affect the observed frequency in the composition.

Simulations of Morris two bidirectionally coupled Morris–Lecar neurons for $C_1 = 1.2$, $C_2 = 1$ that synchronize anti-phase (Fig. S2) with their natural frequency ~ 26 Hz emphasized by the dashed line in the Fig. 3 of the main article. The summed signal exhibits doubled frequency ~ 52 Hz.

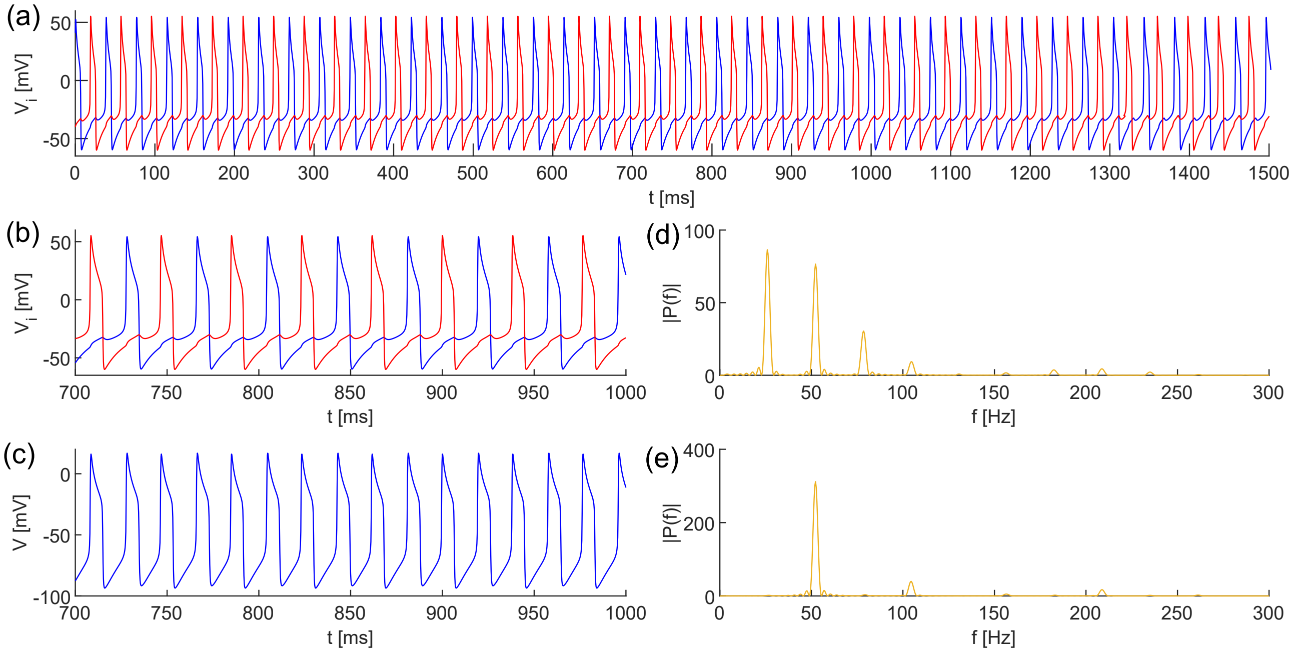

Fig. S2: (a) APS of two bidirectionally coupled Morris–Lecar neurons for $C_1 = 1.2$, $C_2 = 1$, and initial conditions $V_1(0) = 40$, $V_2(0) = -40$, $w_1(0) = w_2(0) = 0.04$ zooming in at the dynamics of (b) V_1 and V_2, (c) $V_1 + V_2$. The remaining parameter values are listed in Table 1 of the main article, $\varepsilon = 0.05$. By comparison of the periodograms corresponding to (d) individual neurons and (e) the composed signal $V = V_1 + V_2$, one can see that this setting leads to a doubled dominant frequency in the composed signal.

More comprehensive bifurcation analysis with respect to parameters C_1 and ε revealed that the anti-phase solution of the system of two coupled interneurons undergoes a supercritical Neimark–Sacker (torus) bifurcation of a limit cycle. This bifurcation causes a loose of the cycle's stability and gives rise to a stable invariant torus in its neighborhood (see Kuznetsov (2023); Wiggins (2003)). This phenomenon is behind the scope of this article; we will publish these results in a forthcoming paper. Nonetheless, as is shown in Fig. S3, the corresponding more complex dynamics remains close to the anti-phase cycle and has, therefore, a similar time course, elucidating the presence of the double dominant frequency in the composed signal $V = V_1 + V_2$, see Fig. 7 of the main article.

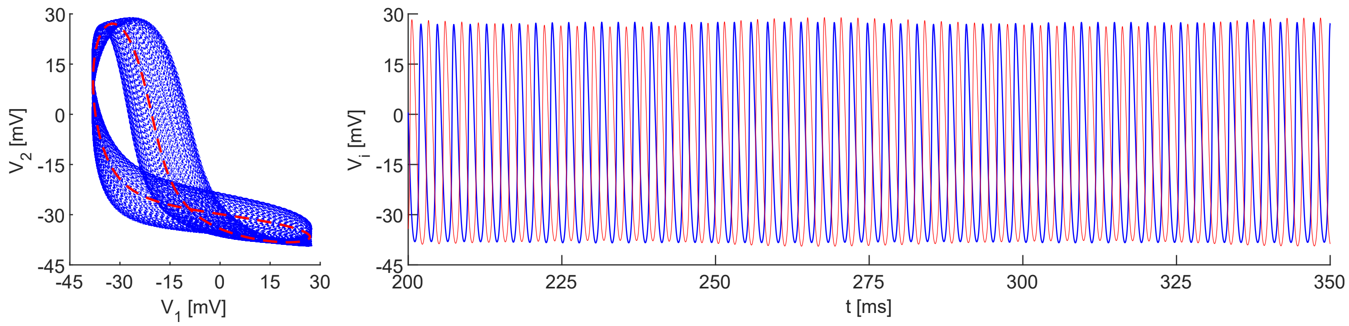

Fig. S3: An example of quasi-periodic orbit arisen through Neimark–Sacker bifurcation of the limit cycle corresponding to the APS in the system of two coupled interneurons for $\varepsilon = 0.04$, $C_1 = 0.99$, and $C_2 = 1$ (see Eq. (17), (19) and Fig. 7 of the main article), $I_{\text{ext}} = 24$, and initial conditions $V_1(0) = 0$, $V_2(0) = -10$, $h_1(0) = 0.3$, $h_2(0) = 0.05$, $n_1(0) = 0.42$, and $n_2(0) = 0.55$. *Left:* plane projections of the orbit (blue dotted line) and unstable (red dashed line) APS onto the state space $V_1 \times V_2$. *Right:* time course of V_1 (blue line) and V_2 (red line) corresponding to the orbit depicted in the left panel.

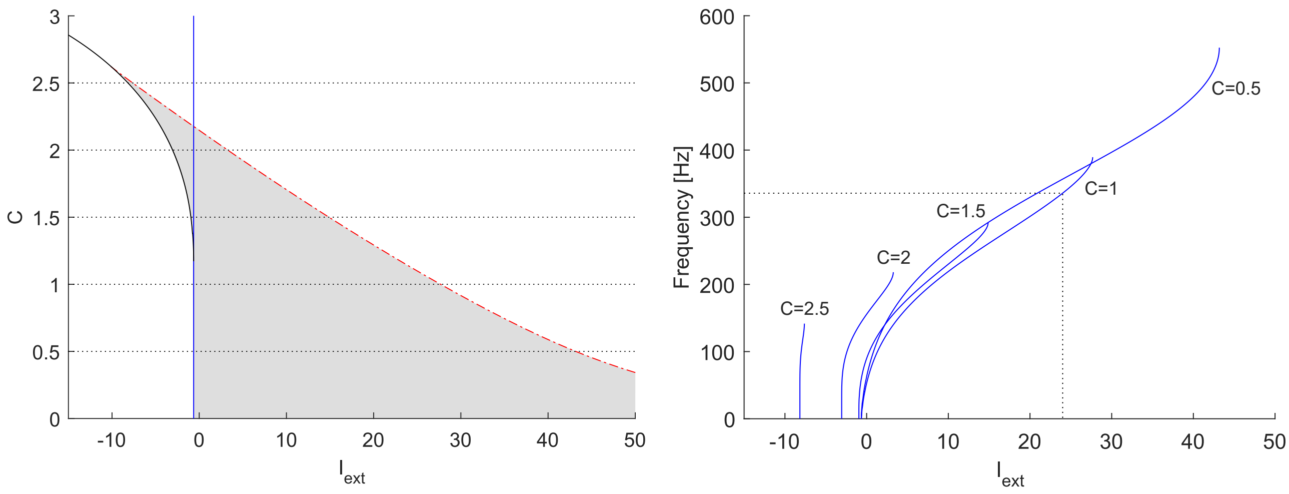

Fig. S4: Interneuron model dynamics with respect to the applied external current I_{ext} and membrane capacitance C. *Left:* The bifurcation diagram shows a tonic spiking region (gray) delineated by a LPC curve (red dash-dotted line), a saddle-homoclinic bifurcation curve (solid black line), and a SNIC bifurcation curve (solid blue line). For simplicity, the other (local) bifurcation curves have been omitted. These result in bistability with a stable equilibrium that will be reported upon in future work. *Right:* The natural firing frequency of the interneuron model as a function of external current I_{ext} for various membrane capacitances C. At $I_{\text{ext}} = 24$ and $C = 1$, the dotted line marks the frequency of $\sim 335\,\text{Hz}$. As the capacitance increases, the frequency range of the oscillation decreases.

REFERENCES

Kuznetsov, Y. A. (2023). *Elements of applied bifurcation theory* (2nd ed., Vol. 112). New York: Springer. doi: 10.1007/978-3-031-22007-4

Wiggins, S. (2003). *Introduction to applied nonlinear dynamical systems and chaos* (2nd ed.). New York: Springer. doi: 10.1007/978-1-4757-4067-7

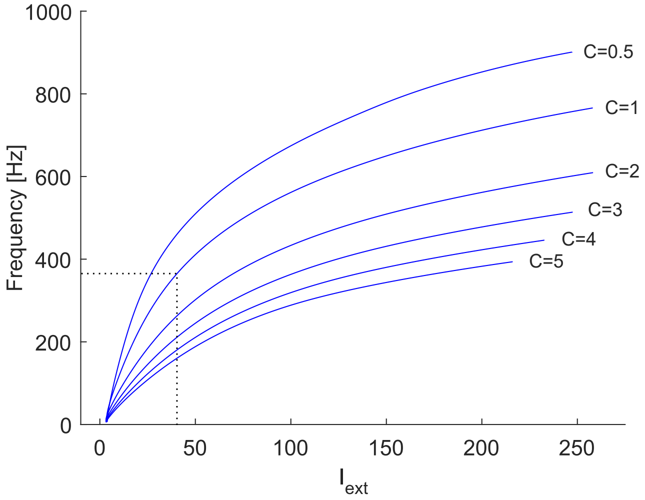

Fig. S5: The natural firing frequency of Destexhe–Paré model as a function of external current I_{ext} for various membrane capacitances C. At $I_{\mathrm{ext}} = 40$ and $C = 1$, the dotted line indicates the frequency of $\sim 360\,\mathrm{Hz}$. As the capacitance increases, only the frequency of the oscillation decreases but does not affect the limit cycle existence.
